# A multiorganism pipeline for antiseizure drug discovery: Identification of chlorothymol as a novel γ-aminobutyric acidergic anticonvulsant

**DOI:** 10.1111/epi.16644

**Published:** 2020-08-14

**Authors:** Alistair Jones, Melissa Barker-Haliski, Andrei S. Ilie, Murray B. Herd, Sarah Baxendale, Celia J. Holdsworth, John-Paul Ashton, Marysia Placzek, Bodiabaduge A. P. Jayasekera, Christopher J. A. Cowie, Jeremy J. Lambert, Andrew J. Trevelyan, H. Steve White, Anthony G. Marson, Vincent T. Cunliffe, Graeme J. Sills, Alan Morgan

**Affiliations:** 1 Institute of Translational Medicine, University of Liverpool, Liverpool, UK; 2 Department of Pharmacy, University of Washington, Seattle; 3 Institute of Neuroscience, University of Newcastle, Newcastle, UK; 4 Neuroscience, Division of Systems Medicine, Ninewells Hospital and Medical School, University of Dundee, Dundee, UK; 5 Department of Biomedical Science, University of Sheffield, Sheffield, UK; 6 Department of Neurosurgery, Royal Victoria Infirmary, Newcastle, UK; 7 School of Life Sciences, University of Glasgow, Glasgow, UK

**Keywords:** drug discovery, epilepsy, GABA, nematode, zebrafish

## Abstract

**Objective:**

Current medicines are ineffective in approximately one-third of people with epilepsy. Therefore, new antiseizure drugs are urgently needed to address this problem of pharmacoresistance. However, traditional rodent seizure and epilepsy models are poorly suited to high-throughput compound screening. Furthermore, testing in a single species increases the chance that therapeutic compounds act on molecular targets that may not be conserved in humans. To address these issues, we developed a pipeline approach using four different organisms.

**Methods:**

We sequentially employed compound library screening in the zebrafish, *Danio rerio*, chemical genetics in the worm, *Caenorhabditis elegans*, electrophysiological analysis in mouse and human brain slices, and preclinical validation in mouse seizure models to identify novel antiseizure drugs and their molecular mechanism of action.

**Results:**

Initially, a library of 1690 compounds was screened in an acute pentylenetetrazol seizure model using *D rerio*. From this screen, the compound chlorothymol was identified as an effective anticonvulsant not only in fish, but also in worms. A subsequent genetic screen in *C elegans* revealed the molecular target of chlorothymol to be LGC-37, a worm γ-aminobutyric acid type A (GABA_A_) receptor subunit. This GABAergic effect was confirmed using in vitro brain slice preparations from both mice and humans, as chlorothymol was shown to enhance tonic and phasic inhibition and this action was reversed by the GABA_A_ receptor antagonist, bicuculline. Finally, chlorothymol exhibited in vivo anticonvulsant efficacy in several mouse seizure assays, including the 6-Hz 44-mA model of pharmacoresistant seizures.

**Significance:**

These findings establish a multiorganism approach that can identify compounds with evolutionarily conserved molecular targets and translational potential, and so may be useful in drug discovery for epilepsy and possibly other conditions.

## 1 | INTRODUCTION

Currently approved antiseizure drugs (ASDs) can provide effective seizure control in around two-thirds of patients. However, the remaining one-third of patients have what is termed drug-resistant, or pharmacoresistant, epilepsy, where seizures cannot be effectively controlled by available ASDs.^1,2^ Therefore, there is a substantial unmet clinical need for new therapeutic compounds for epilepsy. Traditionally, rodent seizure models have been used to evaluate potential ASDs.^3^ Promising compounds are initially assessed for any ability to protect against acute seizure induction through either subcutaneous administration of the convulsant pentylenetetrazol (PTZ) or through transcorneal electrical stimulation in an acute maximal electroshock (MES) or 6-Hz seizure test.^4,5^ For the most part, this approach has been successful in identifying new therapeutic drugs and has provided insights into the molecular mechanism of action of promising agents.^6^ However, ethical concerns, financial costs, and the labor-intensive nature of drug testing in rodents reduce their practicality for early stage, high-throughput compound screening. Furthermore, the proportion of medically refractory cases has remained fairly constant for almost 30 years,^1^ suggesting that epilepsy drug discovery might benefit from using different animal models for frontline ASD screening.

As a result, attention has turned in recent years to using simpler, nonmammalian model organisms for epilepsy research.^7,8^ The fruit fly, *Drosophila melanogaster*, and the zebrafish, *Danio rerio*, are well-established animal models that have been successfully used for high-throughput anticonvulsant drug screening.^9,10^ Several studies have established *D rerio* as a powerful vertebrate alternative to rodent-based drug discovery.^7,11–14^ Zebrafish take up minimal space, have a short generation time, and are amenable to automated motion tracking technology that facilitates high-throughput pharmacological screening platforms. Zebrafish embryos from as young as 2 days postfertilization (dpf) develop a concentration-dependent increase in locomotor activity upon PTZ exposure.^15^ This aberrant locomotion can be coupled to other seizure-related phenotypes seen in higher organisms, including interictal discharges and transcription of neuroprotective immediate-early genes, such as *c-fos*.^16^ *c-fos* activity has been used as the basis for high-throughput screening for compounds able to reduce seizurelike activity, as it can be easily visualized in *D rerio* embryos through whole-mount in situ hybridization.^11^

Modeling epilepsy in the nematode worm, *Caenorhabditis elegans*, is still in its infancy in comparison to zebrafish.^7^ Nevertheless, mutations in various *C elegans* genes have been shown to cause spontaneous seizurelike convulsions, or increased susceptibility to such behaviors, when exposed to PTZ, electroshock treatment, or heat,^17–22^ consistent with higher organisms. Furthermore, various approved ASDs have been shown to ameliorate these seizurelike behaviors,^17,21^ suggesting that *C elegans* has untapped potential for epilepsy drug discovery. In particular, the ease of genetic manipulation and maintenance of genetically modified strains in comparison to other animal models means that *C elegans* is especially powerful for discovering ASD mechanisms of action via chemical-genetic approaches.^21,23^

Each model organism used in epilepsy research has its own strengths and weaknesses. We reasoned that combining the strengths of multiple in vivo animal models with in vitro brain tissue electrophysiology would create a pipeline approach for discovering novel ASDs and their mechanisms of action. We demonstrate this approach here, using *D rerio* to identify the hit compound, chlorothymol, from a compound library screen. We show that chlorothymol has anticonvulsant properties in both *D rerio* and *C elegans*. Subsequently, using a chemical-genetic screen, we identify chlorothymol’s molecular target in *C elegans* as the γ-aminobutyric acid type A (GABA_A_) receptor subunit, LGC-37, and confirm this GABAergic mechanism of action using electrophysiological recording from mouse and human brain slices. Finally, we validate this novel drug screening approach through the evaluation of chlorothymol in a battery of well-established preclinical acute and chronic seizure models in mice, which have been instrumental to the identification of all clinically available ASDs to date.^3^ Of note, chlorothymol demonstrated marked efficacy in the 6-Hz 32-mA and 44-mA pharmacoresistant seizure tests, further confirming the utility of a multispecies drug screening approach for novel ASD discovery.

## 2 | MATERIALS AND METHODS

### 2.1 | Materials

All materials were from Sigma-Aldrich unless stated otherwise. The Johns Hopkins Clinical Compound Library (JHCCL)^24^ was supplied by Professor David Sullivan (Baltimore, MD).

### 2.2 | Zebrafish methods

Animals were maintained according to zebrafish standards of care^25^ at 28°C on a 14 hours light/10 hours dark cycle. Compound screening utilized the JHCCL (v1.0).^24^ Embryos were prepared^26^ and *c-fos* in situ hybridization assays were performed^11^ as previously described. Seizurelike activity was measured using the Zebrabox/Zebralab (Zebrabox Viewpoint) automated locomotion tracking system.^11^ All experiments with zebrafish were performed under licence from the UK Home Office and approved by the University of Sheffield Animal Welfare and Ethical Review Body.

### 2.3 | *C elegans* methods

Worms were maintained at 20°C using standard conditions. The *lgc-37;unc-49* double mutant strain was created by crossing the *lgc-37(tm6573)* and *unc-49(e407)* strains and confirmed by genotyping.^21^ PTZ and paralysis assays were performed as previously described.^21^ The UBC_f80M224Q and CBGtg9050C11145D^27^ fosmid constructs were obtained from Source-Bioscience. Three independently derived lines of each transgenic strain were analyzed.

### 2.4 | Brain slice preparation and electrophysiology

Mouse thalamic slices were prepared from C57/Bl6 mice (postnatal day 18–24) of either sex according to standard protocols.^28^ Human tissue was obtained in accordance with ethical approval by the Newcastle and North Tyneside 2 Research Ethics Committee (06/Q1003/51) with clinical governance approval by the Newcastle Upon Tyne National Health Service Foundation Trust (CM/PB/3707). Human neocortical slices were prepared from fresh brain tissue obtained from the margin of resection from patients who underwent neurosurgery for brain tumors. Electrophysiological recording procedures are described in detail in Appendix S1.

### 2.5 | Mouse in vivo seizure tests

Male albino CF-1 mice (Envigo, Harlan) were used as experimental animals. All rodent studies were performed at the University of Washington and approved by the University of Washington Institutional Animal Care and Use Committee and conformed to the ARRIVE Guidelines.^29^

For MES, the electrical stimulus was 50 mA, 60 Hz for 0.2 seconds delivered using equipment similar to that described previously.^30^ Absence of tonic hindlimb extension was considered protected. Mice were challenged with 6-Hz stimulation used to induce seizures at 32 mA and 44 mA for a duration of 3 seconds via corneal electrodes.^31^ Typically, 6-Hz seizures are characterized by an initial momentary stun followed immediately by forelimb clonus, twitching of the vibrissae, and Straub tail. Animals not displaying this behavior were considered protected. The subcutaneous PTZ (scPTZ) assay was conducted using 85 mg/kg PTZ, the convulsant dose of 97% of male CF-1 mice.^31^ Absence of a seizure in the 30-minute observation period was scored as protection. For corneal kindling, mice were kindled to a criterion of five consecutive secondarily generalized seizures (stage 4 or 5, as described by Racine^32^) as previously described.^33,34^ Mice displaying a seizure score ≤ 5 were considered protected. The fixed-speed rotarod test was used to establish minimal motor impairment to calculate median behavior-impairing dose (TD_50_) values.^35^ Compounds were considered toxic if treated mice fell off three times during a 1-minute period.

### 2.6 | Statistical analysis

This was generally performed using GraphPad Prism version 6, using Student *t* tests or analysis of variance (ANOVA) with appropriate corrections for multiple testing. For mouse phenotyping, the dose required to produce the desired end-point in 50% of animals (estimated median effective dose [ED_50_] or TD_50_) in each test, the 95% confidence interval, and the slope of the regression line were calculated by Probit analysis.^36^

Extensive additional detail about all methods is provided in Appendix S1.

## 3 | RESULTS

### 3.1 | Identification of chlorothymol from compound library screening in zebrafish

**FIGURE 1:**
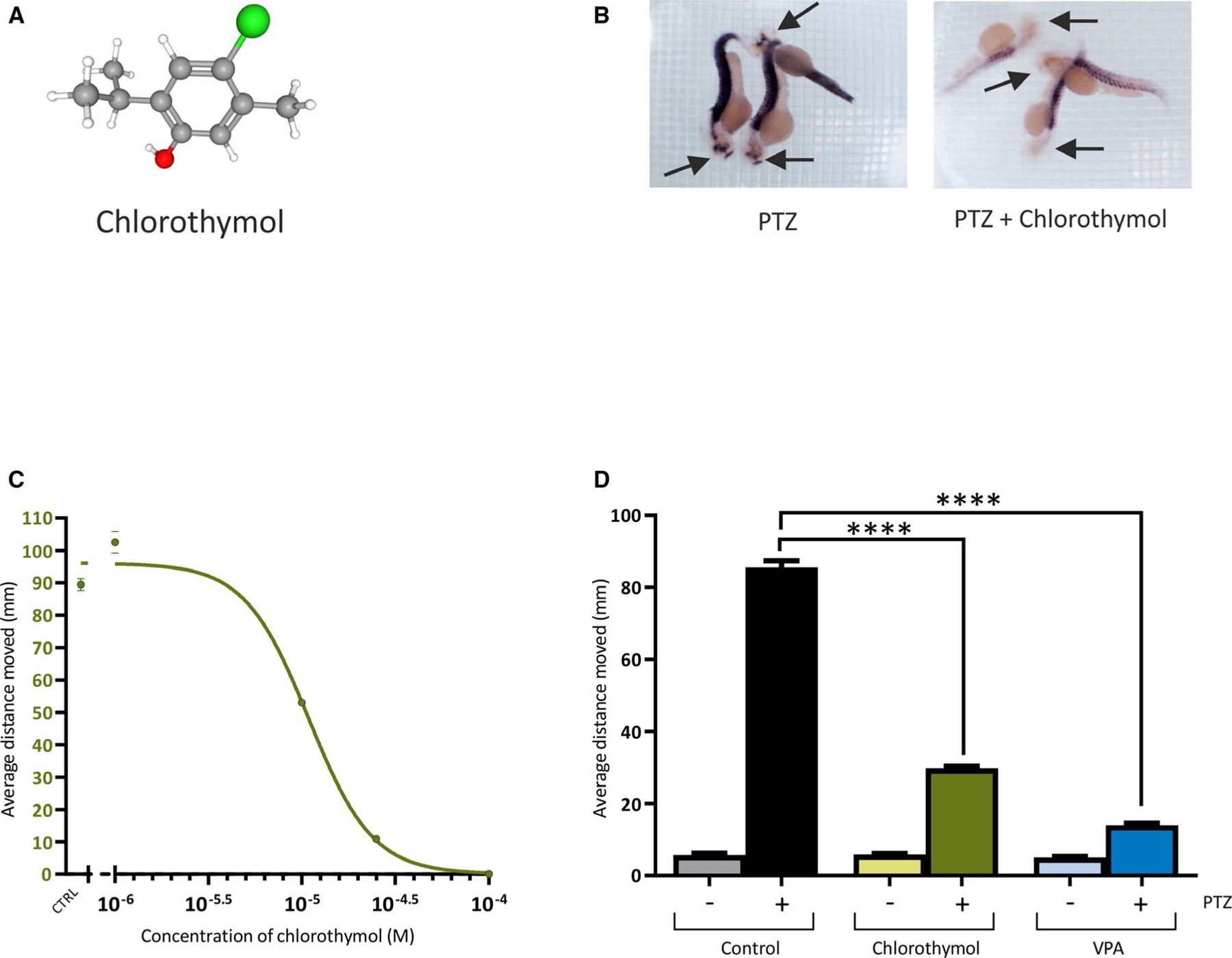
Effects of chlorothymol on pentylenetetrazol (PTZ)-treated zebrafish. A, Chlorothymol structure (carbon = gray, hydrogen = white, oxygen = red, chlorine = green). B, In situ hybridization to detect *c-fos* mRNA in 2 days postfertilization (dpf) *Danio rerio* larvae pretreated with dimethylsulfoxide (DMSO) vehicle only (left panel) or 25 μmol·L^−1^ chlorothymol in DMSO (right panel) before exposure to 20 mmol·L^−1^ PTZ. Arrows indicate the head of each embryo, where strong PTZ-induced *c-fos* expression is suppressed by exposure to chlorothymol. C, Chlorothymol reduces PTZ-induced locomotor behavior in a concentration-dependent manner. The average distance moved by 3-dpf *D rerio* larvae following introduction of 20 mmol·L^−1^ PTZ was recorded over a 1-hour observation period in fish pretreated with a range of chlorothymol concentrations. Data displayed show mean values ± SEM (n = 18 fish per concentration, N = 3 independent experiments). D, Chlorothymol is more potent than the prototype antiseizure drug, valproate (VPA), as it reduced the seizurelike behavior at a 100-fold lower concentration. The average distance moved by 3-dpf *D rerio* larvae following introduction of 20 mmol·L^−1^ PTZ was recorded over a 1-hour observation period in fish pretreated with vehicle only (control), 25 μmol·L^−1^ chlorothymol, or 2.5 mmol·L^−1^ VPA. Data displayed show mean values ± SEM (n = 24 fish per treatment, N = 3 independent experiments). Data were analyzed using one-way analysis of variance with Tukey multiple comparisons test (****P ≤ .0001 compared to PTZ-treated animals)

Treatment of *D rerio* with PTZ induces expression of the transcription factor *c-fos*, an indirect marker of increased neuronal activity and stress.^16^ Compounds that can prevent this upregulation of *c-fos* represent potential antiseizure agents.^11^ We screened the JHCCL of 1690 compounds,^24^ using an in situ hybridization assay to detect *c-fos* transcripts in 2-dpf zebrafish embryos.^11^ Among the hits obtained in this screen was chlorothymol (JHCCL identification number JHU-2077; Figure 1A). Preincubation of 2-dpf embryos with 25 μmol·L^−1^ chlorothymol for 60 minutes before exposure to 20 mmol·L^−1^ PTZ caused a marked reduction in *c-fos* expression (Figure 1B). Subsequent quantitative real-time polymerase chain reaction substantiated these results in 3-dpf larvae, with chlorothymol causing an approximately threefold reduction in PTZ-induced *c-fos* expression (Figure S1). We then set out to determine whether chlorothymol could also ameliorate seizurelike behavior. PTZ can induce several distinct phenotypes in *D rerio*, including an increase in sporadic locomotion often coupled with a “whirlpool” behavior.^11,16^ Using automated tracking software, we measured movement of 3-dpf larvae following application of 20 mmol·L^−1^ PTZ. Preincubation with chlorothymol produced a concentration-dependent reduction of seizurelike locomotion behavior (Figure 1C). At 25 μmol·L^−1^, chlorothymol reduced PTZ-induced movement by approximately threefold, but had little effect on basal locomotion when fish were treated with 25 μmol·L^−1^ chlorothymol alone (Figures 1D and S2). At a higher concentration of 100 μmol·L^−1^, chlorothymol reduced basal locomotion, suggesting that, like many approved ASDs, it may have sedative properties at concentrations above the therapeutic range. The efficacy of 25 μmol·L^−1^ chlorothymol was then compared to that of the clinically approved ASD, sodium valproate (VPA), at a concentration (2.5 mmol·L^−1^) that produces strong anticonvulsant effects in zebrafish but is below the maximum tolerable concentration.^15^ Chlorothymol was significantly more potent against PTZ-induced seizurelike locomotion than VPA, attenuating activity at a 100-fold lower concentration (Figures 1D and S2).

### 3.2 | Chlorothymol reduces PTZ-induced seizurelike activity in *C elegans*

**FIGURE 2:**
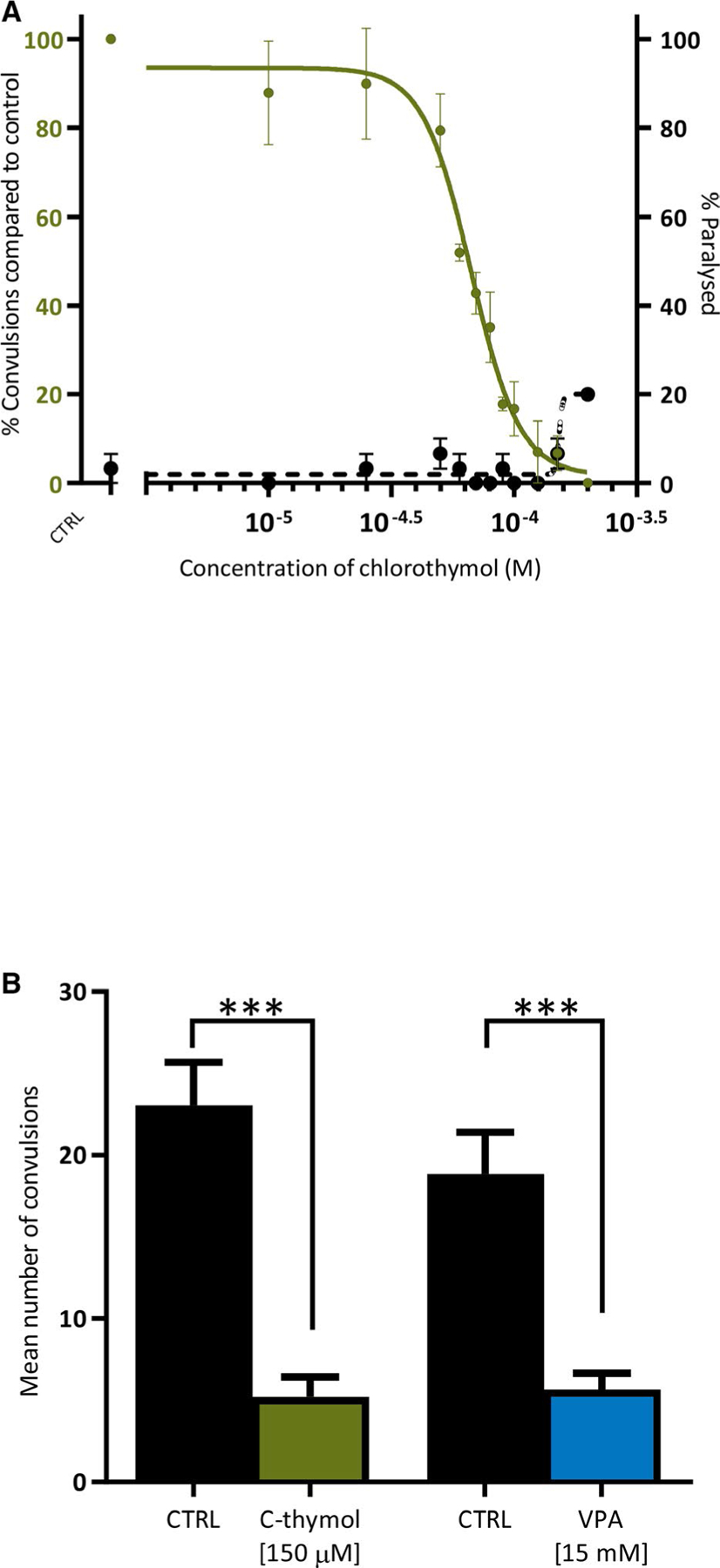
Chlorothymol has anticonvulsant activity in *Caenorhabditis elegans*. A, Chlorothymol reduces pentylenetetrazol (PTZ)-induced convulsions in a concentration-dependent manner. Seizure-prone *unc-49(e407)* mutant worms were preincubated for 15 minutes with a range of chlorothymol concentrations before incubation in the presence (green line) or absence (black dotted line) of 50 mmol·L^−1^ PTZ. The total number of head-bobbing convulsions was measured over a 30-second period and displayed as a percentage of PTZ-treated controls ± SEM (n = 10 worms per concentration, N = 3 independent experiments). B, *C elegans* treated with the optimal anticonvulsant concentration of 150 μmol·L^−1^ chlorothymol determined in A showed a similar level of seizure reduction to the optimal concentration of valproate (VPA; 15 mmol·L^−1^). Data are displayed as mean ± SEM (n = 10 worms per treatment, N = 3 independent experiments) and were analyzed using one-way analysis of variance with Tukey multiple comparison test (***P ≤ .001 compared to respective PTZ-only treatment groups)

To determine whether chlorothymol acts via an evolutionarily conserved target, the drug was tested in a *C elegans* liquid-based assay of seizurelike activity. This involved treating the seizure-prone *unc-49(e407)* strain with PTZ for 15 minutes and then quantifying the number of head-bobbing convulsions over a 30-second period.^21^ The concentration of PTZ used (50 mmol·L^−1^) was previously established as optimal based on concentration-response studies.^21^ Although this PTZ concentration appears high, it is important to note that this is the level in the bathing medium only. The internal concentration experienced by *C elegans* neurons is likely to be substantially lower, as the worm cuticle forms a barrier that greatly reduces the bioaccumulation of most compounds.^37^ Using this method, we again found that pretreatment with chlorothymol could reduce seizurelike activity in a concentration-dependent manner (Figure 2A). At 150 μmol·L^−1^ chlorothymol, PTZ-induced head-bob convulsions were reduced by 93% compared to control, with negligible paralysis observed in the presence or absence of PTZ treatment (Figure 2A; Videos S1 and S2). This demonstrated a therapeutic window where anticonvulsant activity was high whereas motor impairment was minimal. The effect of 150 μmol·L^−1^ chlorothymol was then compared to VPA, having first established the optimal concentration of VPA to be 15 mmol·L^−1^ in this assay (Figure S3). Chlorothymol exhibited a similar level of anticonvulsant activity to VPA in *C elegans* (Figure 2B), but at a 100-fold lower optimal concentration, thus mirroring the activity observed in zebrafish. The combined data from these two simple model organisms therefore suggested an evolutionarily conserved anticonvulsant mechanism of action of chlorothymol.

### 3.3 | Chlorothymol’s molecular target in *C elegans* is the GABA_A_ receptor subunit, LGC-37

**FIGURE 3:**
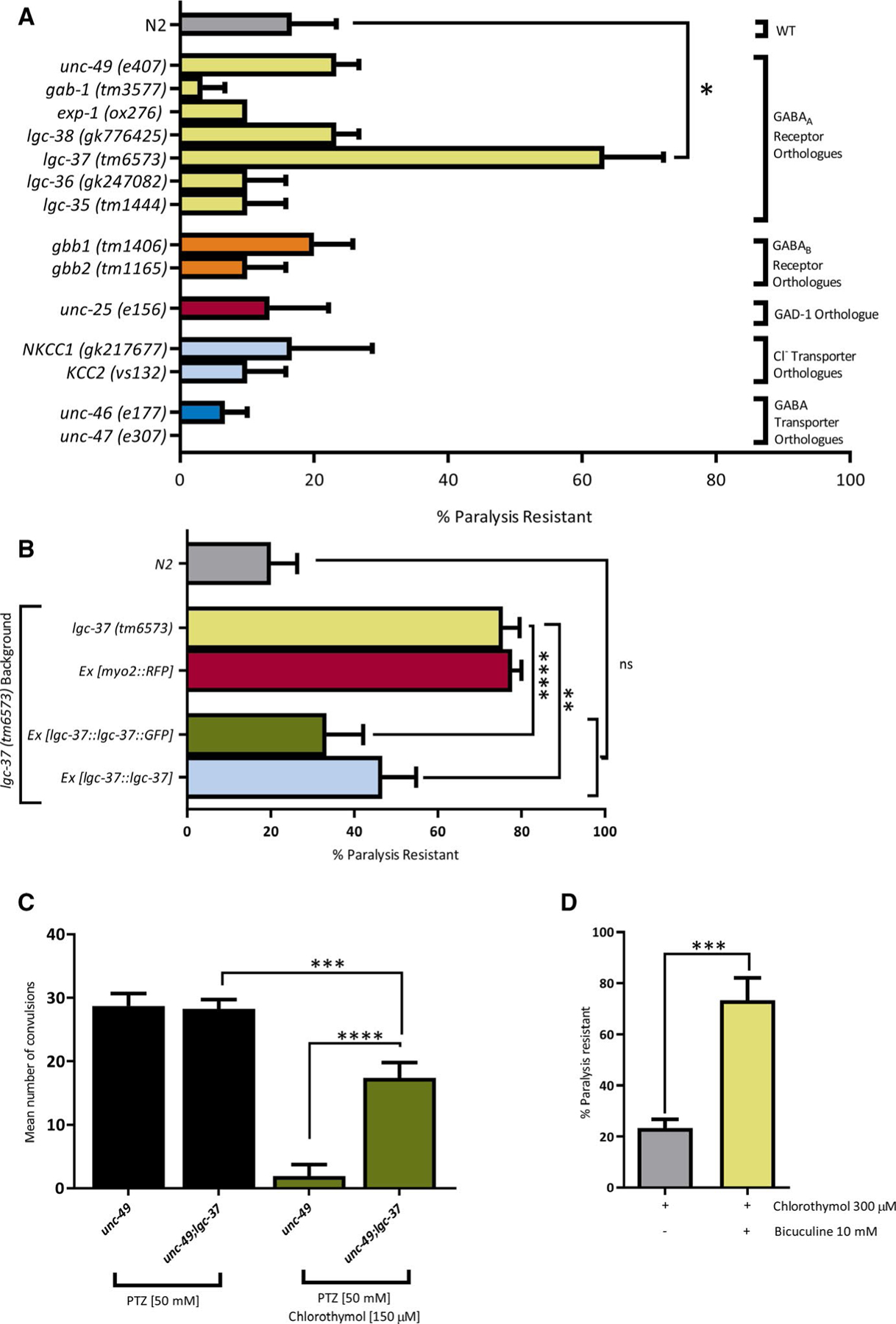
Chlorothymol acts via the *Caenorhabditis elegans* γ-aminobutyric acid type A (GABA_A_) receptor subunit, LGC-37. A, Genetic screen for chlorothymol-resistant mutants. *C elegans* strains with mutations in genes related to GABA function were incubated for 15 minutes in 300 μmol·L^−1^ chlorothymol. The proportion of animals that remained paralyzed over a subsequent 30-second interval was then scored (n = 10 worms per strain, N = 3 independent experiments). The *lgc-37*(*tm6573*) mutant strain was found to be significantly resistant to this paralysis (*P < .05 compared to wild-type [WT] N2 strain). B, Transgenic expression of wild-type *lgc-37* DNA constructs reverses chlorothymol resistance in *lgc-37* mutants. Sensitivity to chlorothymol-induced paralysis in *lgc-37* mutants expressing either green fluorescent protein (GFP)-tagged (*Ex [lgc-37::lgc-37::GFP]*) or untagged (*Ex [lgc-37::lgc-37]*) *lgc-37* genomic clones was not significantly different from wild-type N2 controls, whereas expression of the fluorescent marker alone (*Ex [myo-2::RFP]*) had no effect (n = 15 worms per strain, N = 3 independent experiments; **P < .01, ****P ≤ .0001 compared to the *lgc-37* strain). ns, not significant; RFP, red fluorescent protein. C, Mutation of *lgc-37* reduces the anticonvulsant effect of chlorothymol. Single mutant *unc-49* and double mutant *lgc-37;unc-49* strains were compared in the pentylenetetrazol (PTZ) assay of seizure-related activity. Both strains produced similar levels of PTZ-induced convulsions, but the anticonvulsant effect of chlorothymol was greatly reduced in *lgc-37;unc-49* double mutants (n = 10 worms per treatment, N = 3 independent experiments, ***P < .001, ****P ≤ .0001 compared to the *unc-49* strain). D, The competitive GABA_A_ antagonist, bicuculline (10 mmol·L^−1^) prevents paralysis induced by 300 μmol·L^−1^ chlorothymol (n = 10 worms per treatment, N = 3 independent experiments, ***P < .001 compared to worms treated with chlorothymol only). Data are shown as mean ± SEM and were analyzed using one-way analysis of variance with Tukey multiple comparison test (A-C) or by unpaired *t* test (D)

There are very few published studies of chlorothymol, but it has been suggested that the compound exhibits membrane-modifying and antioxidant properties, as well as effects on GABAergic signaling,^38–40^ which could account for its anticonvulsant properties. As chlorothymol is structurally related to some compounds with GABAergic activity, such as propofol, we performed a chemical-genetic screen in *C elegans* to test the hypothesis that chlorothymol’s in vivo molecular target was a component of the GABA machinery. To this end, we obtained strains containing mutations in known elements of the *C elegans* GABA system. This included seven GABA_A_ receptors, two GABAB receptors, two GABA transporters, two chloride transporters, and the GABA-synthesizing enzyme glutamic acid decarboxylase (Figure 3A). We then screened these strains using the paralysis phenotype caused by exposure to a high concentration (300 μmol·L^−1^) of chlorothymol in the absence of PTZ (Video S3), reasoning that mutations in genes encoding chlorothymol’s molecular targets would confer resistance to chlorothymol-induced paralysis.

This initial screen identified *lgc-37*(*tm6573*) as the only strain that was significantly resistant to chlorothymol-induced paralysis in comparison to control wild-type N2 *C elegans* (63% vs 17% paralysis resistance, respectively). The *lgc-37* gene encodes a ligand-gated ion channel subunit homologous to human GABA_A_ receptors.^27^ According to BLASTP analysis, *C elegans* LGC-37 exhibits similar homology to human GABA_A_ receptor gamma 2, alpha 6, and beta 2 subunits (Figure S4). Although one of these isoforms may represent the true orthologue of LGC-37, it seems more likely that LGC-37 is an evolutionarily ancient GABA_A_ receptor that fulfils the role of more than one of the human isoform subunits. There are no published functional data on LGC-37, but green fluorescent protein (GFP)-tagging studies in *C elegans* have shown it to be expressed in a wide variety of neuronal cell types, and also in muscle.^27^ To confirm that chlorothymol resistance in the *lgc-37*(*tm6573*) strain was due to the *lgc-37* mutation and not some other defect, we attempted to rescue this phenotype by reintroduction of wild-type *lgc-37* (Figure 3B). Fosmids containing the native *lgc-37* gene or a GFP*-*tagged *lgc-37* construct^27^ were microinjected into the gonads of the *lgc-37*(*tm6573*) mutant strain alongside a pharynx-specific red fluorescent protein (RFP) marker to identify transgenic progeny. We found that paralysis resistance could be rescued back to wild type-levels in transgenic worms by both untagged and GFP-tagged *lgc-37* constructs, whereas the RFP marker alone had no effect (Figure 3B).

Having confirmed that resistance to chlorothymol-induced paralysis is mediated by LGC-37, we set out to determine whether LGC-37 was also the target of chlorothymol’s anticonvulsant activity. To this end, we crossed the *lgc-37*(*tm6573*) strain with the *unc-49*(*e407*) strain used for the seizure assays to create a double mutant strain harboring both *lgc-37* and *unc-49* alleles. The double mutant was indistinguishable from the single *unc-49* mutant in terms of the number and frequency of convulsions caused by PTZ treatment. However, the ability of 150 μmol·L^−1^ chlorothymol to prevent such PTZ-induced convulsions was greatly reduced in the double mutant, confirming *LGC-37* as the main target of chlorothymol’s anticonvulsant activity (Figure 3C). Nevertheless, the effect of chlorothymol was not entirely abolished in the *lgc-37;unc-49* double mutant, suggesting that the compound may also have minor activity on other molecular targets.

To validate our chemical-genetic identification of chlorothymol’s in vivo mechanism of action, we took an alternative pharmacological approach using the GABA_A_ competitive antagonist, bicuculline. Treatment of wild-type N2 worms with 10 mmol·L^−1^ bicuculline methiodide reduced chlorothymol-induced paralysis from 77% to 27%, thus confirming chlorothymol’s pro-GABAergic action (Figure 3D, Video S4). Finally, to ensure that chlorothymol’s anticonvulsant activity was not due to sedative effects associated with some GABAergic drugs, we performed a concentration-response experiment using the general anesthetic etomidate, a positive modulator of GABA_A_ receptors. No significant anticonvulsant effects of etomidate were detected even at the highest concentration tested (400 μmol·L^−1^), where around 50% of worms became paralyzed by the drug (Figure S5). Taken together, these data indicate that chlorothymol’s anticonvulsant mechanism of action in *C elegans* requires the GABA_A_ receptor subunit, LGC-37.

### 3.4 | Chlorothymol enhances phasic and tonic GABA_A_ receptor signaling in mammalian brain

**FIGURE 4:**
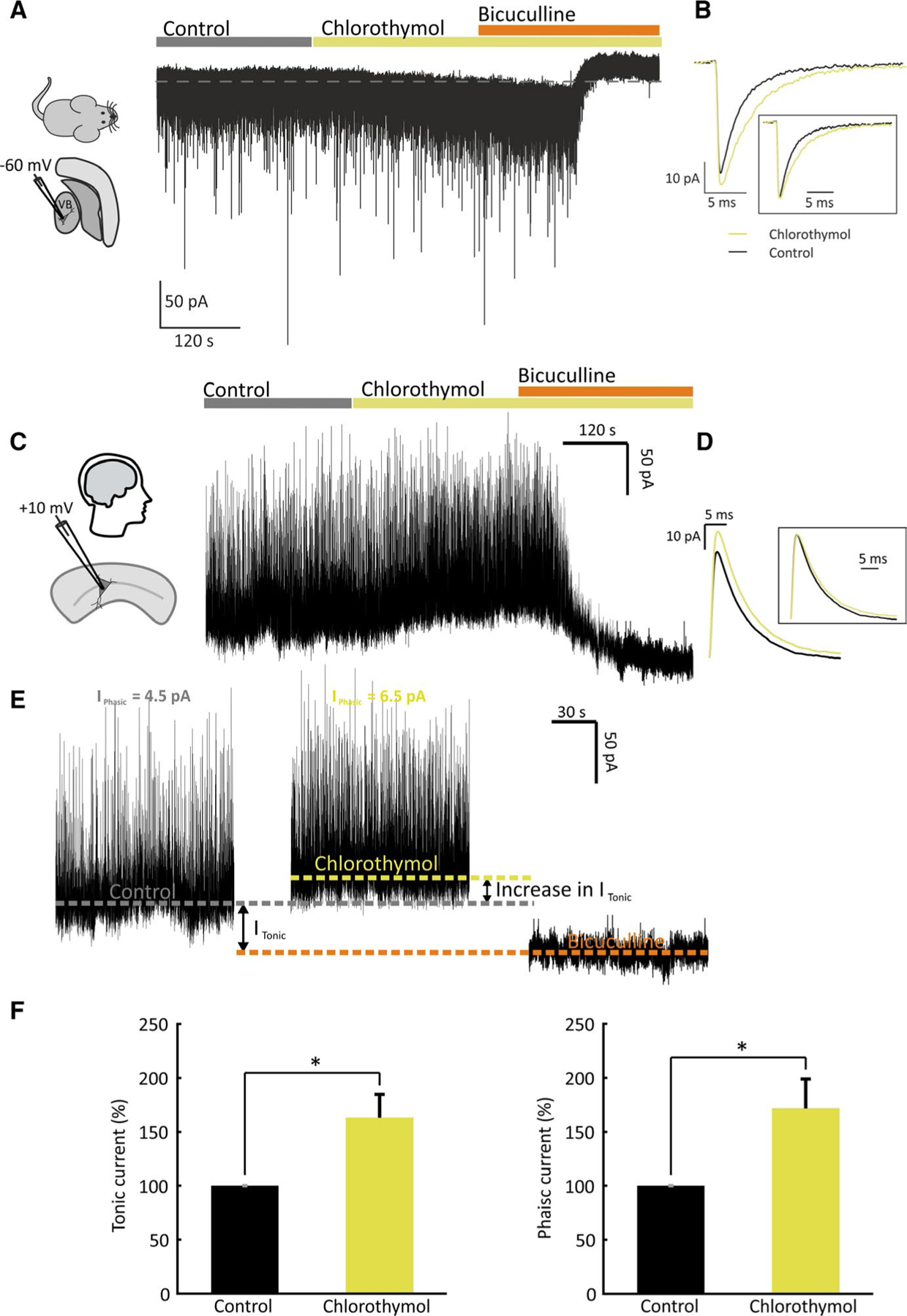
Chlorothymol increases tonic and phasic γ-aminobutyric acidergic currents in mouse and human brain slices. A, Example whole-cell recording from a mouse thalamic ventrobasal (VB) neuron. Inhibitory currents were isolated by holding the cell at −60 mV in voltage clamp mode in the presence of 2 mmol·L^−1^ kynurenic acid and 0.5 μmol·L^−1^ tetrodotoxin. Bath application of 54 μmol·L^−1^ chlorothymol induced an inward shift in the holding current, consistent with an enhancement of the resident tonic inhibitory conductance (revealed by subsequent application of 30 μmol·L^−1^ bicuculline). B, Superimposed miniature inhibitory postsynaptic current (mIPSC) averages obtained before and after chlorothymol application (from the same cell shown in A). The boxed inset illustrates the averaged chlorothymol current normalized to the control peak amplitude. C, Example whole-cell recording from a human cortical neuron. Inhibitory currents were isolated by holding the cell at +10 mV in voltage clamp mode. Chlorothymol (54 μmol·L^−1^) and bicuculline (10 μmol·L^−1^) were sequentially applied to test their effects on inhibitory currents. D, Superimposed mIPSC averages obtained before and after chlorothymol application (from the cell shown in C). The boxed inset shows averaged chlorothymol current normalized to the control peak amplitude. E, Expanded timepoints from the recording in C under control, chlorothymol, and bicuculline conditions. Colored dotted lines represent the holding current under these three conditions. Bicuculline blocks both phasic (*I*_phasic_) and tonic (*I*_*t*onic_) currents and hence can be used to calculate *I*_tonic_ in control conditions as the difference between the holding current under control and bicuculline conditions. The increase in *I*_tonic_ induced by chlorothymol was calculated as the difference in holding current between chlorothymol and control. *I*_phasic_ was calculated by averaging the traces after subtraction of the holding current (see Materials and Methods for full details). F, Population data showing that chlorothymol increased both *I*_tonic_ and *I*_phasic_ in human cortical neurons. Data are shown as mean ± SEM (n = 6 brain slices) and were analyzed by paired *t* tests (*P < .05)

To investigate the mechanism by which chlorothymol interacts with the GABA_A_ receptor, and to test whether this interaction is preserved in the mammalian brain, we performed a series of electrophysiological experiments using both mouse and human neurons. We first tested the effect of chlorothymol on GABA_A_ receptor signaling using mouse thalamocortical brain slices. Ventrobasal (VB) neurons in these preparations exhibit GABAergic phasic and tonic inhibition mediated by synaptic α1β2γ2 and extrasynaptic α4β2δ GABA_A_ receptors, respectively.^28,41–43^ We isolated phasic and tonic GABAergic currents by performing whole cell voltage-clamp recordings in the presence of kynurenic acid to block glutamate receptors and tetrodotoxin to block action potentials. We used a high intracellular chloride solution and held the neurons at −60 mV; in this configuration, phasic GABAergic currents are recorded as negative deflections in the traces and changes in the holding current represent changes in tonic GABAergic currents (Figure 4A). Application of 54 μmol·L^−1^ chlorothymol increased the holding current to 180% of that seen under control conditions (Figure S6), consistent with an increase in the tonic GABAergic inhibitory conductance displayed by VB neurons. This was reversed by subsequent application of 30 μmol·L^−1^ bicuculline, confirming that the measured changes in holding current were mediated by GABA_A_ receptors (Figure 4A). In addition, chlorothymol application increased phasic GABAergic signaling by significantly prolonging the decay phase and increasing the peak amplitude of VB miniature inhibitory postsynaptic currents (mIPSCs; Figure 4B). This resulted in an increase in the average charge transferred per mIPSC to 180% of control (where control is 100%, ie, an 80% increase over the control; Figure S6). Collectively, these experiments demonstrate that chlorothymol exerts positive effects on GABA_A_ receptor isoforms that are commonly expressed at synaptic (α1/γ2-containing) and extrasynaptic (δ-containing) locations in the mouse brain, thus enhancing phasic and tonic GABAergic currents.

To investigate the translational potential of chlorothymol, we tested whether chlorothymol could also enhance GABAergic currents in human neurons. We used human cortical brain slices prepared from the resection margin from patients who required neurosurgical interventions for brain tumors. To isolate GABAergic inhibitory currents, without perturbing the network activity by using glutamate blockers, we performed patch-clamp recordings in voltage clamp mode and held the membrane potential at +10 mV, close to the reversal potential for glutamate (Figure 4C; see Jirsa et al^44^). Using this configuration, phasic GABAergic currents are recorded as positive deflections in the traces, and changes in holding current represent changes in tonic GABAergic currents (Figure 4C). Phasic and tonic inhibitory currents (*I*_Pha_ and *I*_Ton_) were separated based on a modification of a published method.^45^ Chlorothymol (54 μmol·L^−1^) and bicuculline (10 μmol·L^−1^) were sequentially applied, which allowed us to record the percentage change in *I*_Ton_ and *I*_Pha_ induced by chlorothymol. As illustrated in Figure 4C–F, chlorothymol increased *I*_Ton_ and *I*_Pha_ to 163% and 172% of control, respectively (where control is 100%; ie, 63% and 72% increases over the control). Conversely, bicuculline significantly reduced *I*_Pha_ to 15.7% of control and blocked *I*_Ton_, as illustrated by the reduction in holding current. Analysis of mIPSCs revealed that chlorothymol significantly increased mIPSC amplitude and charge transfer (Figure S7). The data from human cortical neurons were remarkably consistent with the effects of chlorothymol on GABAergic currents recorded in mouse thalamocortical neurons. Taken together, these results demonstrate that chlorothymol acts by increasing GABAergic signaling, and this mechanism of action is preserved in both mouse and human neurons.

### 3.5 | Chlorothymol exhibits anticonvulsant activity in mouse acute and chronic seizure models

As a final validation step in our drug discovery pipeline, the effect of chlorothymol was investigated in a battery of well-established, preclinical mouse seizure paradigms that have been instrumental to the identification of all ASDs currently available in the clinic.^3^ The tests included the MES test, the 6-Hz test at 32 and 44 mA, the scPTZ test, and the corneal kindled mouse model. In addition to defining the potential for antiseizure efficacy, we also sought to define the potential for motor-impairing effects by quantifying the TD_50_ using the rotarod test.^35^ Chlorothymol was found to have a TD_50_ of 151 mg/kg when administered intraperitoneally (IP) 1 hour prior to testing. The IP administration interval of 1 hour prior to testing was found to be the time of peak effect for the compound, for all of the tests shown; however, chlorothymol demonstrated rapid anticonvulsant activity that was maintained for at least 4 hours. Chlorothymol was found to exert potent and sustained activity against 6-Hz focal psychomotor seizures, at both 32 mA (ED_50_ = 18.0 mg/kg) and 44 mA current intensities (ED_50_ = 66.2 mg/kg), at doses that were devoid of behavioral toxicity, yielding protective index (PI; TD_50_/ED_50_) values of 8.4 and 2.3, respectively (Table 1). Chlorothymol was less potent in the scPTZ test, but nevertheless reduced clonic seizures up to the end point of 0.5 hour following PTZ administration (ED_50_ = 118 mg/kg; PI = 1.3). It also blocked the tonic-extension component of MES seizures (ED_50_ = 136 mg/kg), albeit with a low PI of 1.1.

Having established efficacy in acute seizure models, corneal kindled mice were then used to test whether chlorothymol was also effective in a chronic network hyperexcitability assay.^32^ Chlorothymol was found to dose-dependently reduce kindled seizure severity in corneal kindled mice, at a calculated ED_50_ of 115 mg/kg (PI value of 1.3; Table 1 and Figure S8). Importantly, this finding demonstrates that chlorothymol exerts anticonvulsant activity in a chronically hyperexcitable rodent model. Finally, VPA was used as a positive control in the 6-Hz 32-mA and MES tests, as these produced chlorothymol’s highest and lowest PI values, respectively. Although VPA provided better protection in the MES test, chlorothymol displayed greater PIs than VPA in the 6-Hz 32-mA and 44-mA tests (PI values of 8.4 and 2.8, respectively). Therefore, chlorothymol has protective activity in several well-validated mouse acute and chronic seizure models, including the mouse 6-Hz 44-mA model of pharmacoresistant seizures. Furthermore, the anticonvulsant efficacy of chlorothymol is consistent with the broad spectrum ASD, VPA.

## 4 | DISCUSSION

**FIGURE 5:**
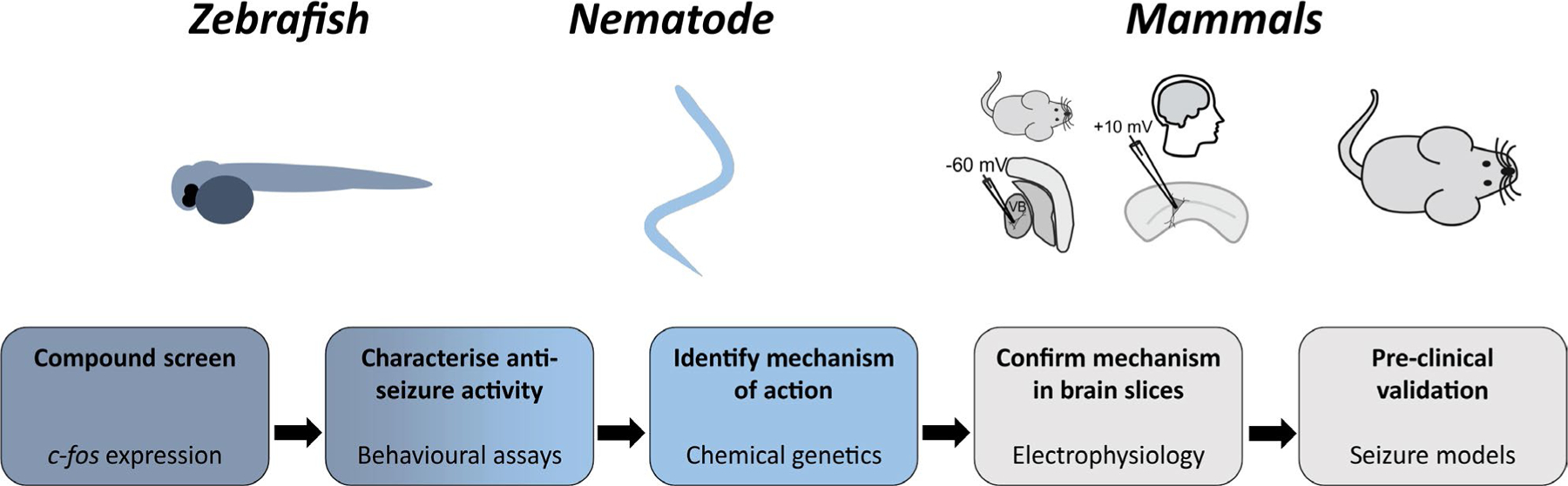
A multiorganism pipeline for antiseizure drug discovery. The schematic diagram illustrates the different stages used in our approach. VB, ventrobasal

New therapeutic options are urgently needed for the large number of people with pharmacoresistant epilepsy.^1^ However, developing new drugs typically takes >20 years, and much of this time is spent on early drug discovery and translational science rather than on later clinical studies.^46^ The multiorganism pipeline approach described here (Figure 5), which exploits the power of simple model organisms for pharmacological and genetic screening prior to validation in more complex mammalian systems, can potentially accelerate such early preclinical studies. We chose zebrafish for the first stage of the pipeline (the initial compound library screen), as this organism has demonstrated utility in pharmacological screens for novel ASDs.^7,11–14^ A cornerstone of our strategy is that hit compounds must then be validated in an evolutionarily distant organism, to avoid potential species-specific effects. We chose *C elegans* for this second stage, because of its facile molecular genetics and large library of viable mutant strains. This confers the additional advantage of enabling much faster chemical-genetic identification of a drug’s mechanism of action than could be achieved in other animal models. These features allowed us to confirm chlorothymol’s anticonvulsant activity and to reveal its molecular target as a GABA_A_ receptor subunit. Nevertheless, various other invertebrate model organisms could be used for these early stages, for example *Drosophila*.^9^ The next stage in our pipeline was to establish that hit compound activity and molecular mechanism are conserved in mammalian systems, including humans, and therefore have translational potential. For this, we used mouse and human brain slice electrophysiology to confirm the GABAergic action of chlorothymol in neurons. Finally, therapeutic activity should be demonstrated in a widely accepted and preferably clinically validated in vivo mammalian model. Our finding that chlorothymol confers antiseizure efficacy in several clinically validated and/or well-established mouse seizure models—including the MES, corneal kindled mouse, and 6-Hz 44-mA models of pharmacoresistant seizures—therefore provides the proof of principle that this multiorganism pipeline approach can reveal novel anticonvulsants with clear therapeutic potential. One drawback of our use of human brain to validate translational potential is the difficulty of obtaining tissue for experiments. This represents a bottleneck in the approach described here, but could alternatively be performed as the last stage in the pipeline after preclinical testing in rodents. Although our chemical screen identified a molecule with a target (the GABA_A_ receptor) that is already established in the treatment of epilepsy, the inclusion in our pipeline of *C elegans*, which lacks voltage-gated sodium channels (the most common target of current ASDs), should increase the chance of finding ASDs with novel targets compared to existing approaches that do not include this animal model. In the future, the ability to rapidly create models of specific genetic epilepsy syndromes in both fish and worms using CRISPR could be used to develop drug screens targeted at specific causative variants in candidate genes.^7^ Moreover, the pipeline approach is applicable to many different disorders and so may be of more general interest beyond the clinical indication of epilepsy.

Although little has been published on chlorothymol, it has been suggested to possess GABAergic, membrane-modifying, and antioxidant properties.^38–40^ Our results using genetic and pharmacological approaches in worm, mouse, and human systems strongly indicate that chlorothymol’s anticonvulsant action occurs via positive modulation of GABA_A_ receptors. This is consistent with the importance of the GABAergic system in epilepsy; variants in GABA_A_ receptor subunits are a common cause of genetic epilepsies,^47^ and various approved ASDs act by GABA potentiation.^48^ Although a variety of GABA_A_ receptor modulating compounds are already available, there are some unique properties of chlorothymol that suggest it may have translational potential for epilepsy indications. First, it is structurally unrelated to any clinically approved ASD. Second, chlorothymol’s therapeutic profile in several mouse seizure assays differentiates it from most GABAergic ASDs, in particular the benzodiazepines that typically show highest potency in the scPTZ test.^3^ This is clearly seen for diazepam using either the same CF-1 mice from Envigo used in this study^49^ or mice from Charles River (available on the National Institute of Neurological Disorders and Stroke Panache Database https://panache.ninds.nih.gov). In contrast, chlorothymol did not demonstrate significant activity at nonimpairing doses in the scPTZ test, but did confer significant anticonvulsant activity in the 6-Hz 44-mA model of drug-resistant focal seizures. Hence, although further work is required, chlorothymol (or derivates thereof) has potential applications, not only in epilepsy, but also in other indications where GABA_A_ receptor modulators are used clinically, such as anxiety.

Chlorothymol is inexpensive (less than $2 per gram) and very stable (we have observed no loss in anticonvulsant activity upon storage for >18 months). However, the only report of its clinical use that we have found dates from 1933, where its potential application as a topical antiseptic in obstetrics is described.^50^ The data shown here that high doses of chlorothymol cause motor impairment in mice, fish, and worms suggest that this could be an issue for clinical applications. Comprehensive behavioral assays in addition to the simple rotarod test would therefore be desirable to gain more information on the tolerability and safety of chlorothymol, for example, the habituated open field test.^49^ Nevertheless, a therapeutic window was found in all three model organisms where anticonvulsant effects were observed with minimal sedation, suggesting that the same may be achievable in humans with carefully controlled dosing regimens. Several currently prescribed ASDs (notably VPA) also have very narrow protective indices in animal models^3^ and liability to adverse effects at high doses in humans. In the short term, chlorothymol may have direct clinical applications for status epilepticus, which currently uses benzodiazepines as a first-line treatment to potentiate GABA_A_ receptors and where sedation is less of an issue.^51^ Chlorothymol may therefore have promise as an alternative to benzodiazepines or as a therapy for benzodiazepine-resistant status epilepticus. In the longer term, medicinal chemistry approaches using chlorothymol as a starting scaffold could be used to develop structurally related next generation compounds with increased anticonvulsant activity, reduced sedative effects, and enhanced selectivity at GABA_A_ receptor subtypes. Our pipeline approach would be invaluable for such hit-to-lead optimization, as the bulk of the screening process could be performed quickly and cheaply using frontline fish and worms, with subsequent validation of lead compounds in the more complex and physiologically relevant rodent and human systems.

## Supplementary Material

Video 3

Video 4

Video 2

Video 1

Supporting Material

## Figures and Tables

**TABLE 1:** Effect of chlorothymol and VPA in mouse seizure models

Test	Chlorothymol		VPA	
	ED_50_, mg/kg (95% CI)	PI, ED_50_/TD_50_	ED_50_, mg/kg (95% CI)	PI, ED_50_/TD_50_
6 Hz, 32 mA	18.0 (6.8–28.2)	8.4	139 (92.4–197)	2.8
6 Hz, 44 mA	66.2 (45.8–102)	2.3	289 (242–384)^a^	1.3^a^
Subcutaneous PTZ	118 (83.4–144)	1.3	305 (212–403) ^a^	1.3^a^
Maximal electroshock	136 (106–166)	1.1	213 (138–274)	1.8
Corneal kindled	115 (78.1–205)	1.3	174 (135–208) ^a^	2.2^a^
Rotarod, TD_50_	151 (126–191)		390 (382–396)	

*Note:* At least eight mice were used for each test dose necessary to define an ED_50_/TD_50_ at the previously determined time to peak effect of each compound (1 hour for chlorothymol, 15 minutes for VPA).

Abbreviations: CI, confidence interval; ED_50_, estimated median effective dose; PI, protective index; PTZ, pentylenetetrazol; TD_50_, median behaviorally impairing dose; VPA, valproic acid.

a Data are from the PANAChE database (National Institute of Neurological Disorders and Stroke: https://panache.ninds.nih.gov/ChemDetail.aspx?CHEM_ID=8).

## Key Points

- Compound library screening in zebrafish identifies chlorothymol as an anticonvulsant
- Genetic screening in nematodes reveals that chlorothymol acts on GABA_A_ receptors
- Electrophysiological ecording from mouse and human neurons confirms chlorothymol’s GABAergic mechanism of action
- Chlorothymol is protective in several mouse seizure assays, including the 6-Hz 44-mA model of pharmacoresistant seizures
