# Supplementary material for: A multiorganism pipeline for antiseizure drug discovery: Identification of chlorothymol as a novel γ-aminobutyric acidergic anticonvulsant
Source: Epilepsia. Author manuscript; Available in PMC 2023 Dec 29. (PMC10756143; doi:10.1111/epi.16644)
Supplement: Supporting Material [file NIHMS1946003-supplement-Supporting_Material.pdf]

Methods

Study design

The objective of this study was to develop a pipeline approach to identifying new anticonvulsants based on the sequential use of multiple organisms. Initial compound library screening was performed using a visual *in situ* hybridization assay in *Danio rerio* larvae, followed by qPCR validation and characterization using assays of acute PTZ-induced seizure-like behaviour. *Caenorhabditis elegans* was then used to confirm anticonvulsant activity in an evolutionarily distant animal model and to identify potential mechanisms of action by chemical-genetic screening for drug-resistant mutants. Confirmation of mechanism of action in mammals was then undertaken by electrophysiological recording from both mouse and human brain slices *in vitro*. Finally, pre-clinical validation was performed using a range of well-established mouse *in vivo* seizure assays. Sample sizes for mouse in vivo experiments were calculated using power analysis. Eight animals were used per group, which gives 80% power at 95% significance to detect a 1.5 standard deviation difference between groups. Sample sizes for fish, worm and brain slice experiments were based on published studies from our laboratories that have demonstrated statistically significant effects of pharmacological agents. The number of experimental units per experiment and the number of independent replicate experiments performed are indicated below and also in the figure legends. No data were excluded. Investigators were blind as to the chemical identity of the compounds in the initial library screen. Blinding was not required for zebrafish experiments, where data recording and analysis is computer-based and not influenced by the human experimenter. Similarly, the worm genetic screen is, by its very nature, unbiased and so blinding was not relevant. Electrophysiological experiments were performed by two independent labs and both

labs were blinded to each other doing the work, although experiments in each individual lab were not conducted blind. For the mouse studies, with the exception of the corneal kindled mouse model, all in vivo seizure tests were assessed with a binary “yes/no” scoring system using well-established evaluation criteria – they do not appear on a gradient that could lead to subjective interpretation and so blinding was not deemed necessary. Acute seizure testing was conducted with secondary, simultaneous verification of assigned seizure score by a trained investigator (MB-H) or other technical staff, whenever possible. For the corneal kindling model, the ED_{50} was calculated by two independent investigators (AJ and MB-H) using a binary “protected/not protected” scoring system based on the modified Racine seizure score.

Materials

All materials were obtained from *Sigma Aldrich* (Poole UK) unless stated otherwise in the text.

The Johns Hopkins Clinical Compound Library (version 1.0) was kindly supplied by Professor David Sullivan, of the Johns Hopkins School of Medicine (Baltimore, USA).

Animal maintenance

Zebrafish were maintained according to the zebrafish standards of care[1] at 28°C on a 14 hours light/10 hours dark cycle. Zebrafish eggs were collected at the beginning of each light cycle. Eggs were stored in E3 medium (15 mM NaCl, 0.5 mM KCl, 1 mM $CaCl_2$, 1 mM $MgSO_4$, 1.5 mM KH_2PO_4, 0.05 mM Na_2HPO_4, 0.7 mM $NaHCO_3$) with 1% methylene blue per litre and used at 2, 3 or 4 days post-fertilisation (dpf).

C. elegans were maintained at 20°C on standard nematode growth medium (NGM; 1 mM $CaCl_2$, 1 mM $MgSO_4$, 25 mM KH_2PO_4, 5 µg/mL cholesterol, and in w/v 2% agar, 0.25%

peptone, and 0.3% NaCl). Each 60-cm plastic petri dish plate contained 10 ml NGM and was seeded with 50 µl OP50 (*E. coli*) as a food source. The *lgc-37;unc-49* double mutant strain was created by crossing the *lgc-37*(*tm6573*) and *unc-49*(*e407*) strains and was confirmed by PCR genotyping, with additional restriction fragment length polymorphism analysis for the *unc-49(e407)* point mutation[2]. All strains were sub-cultured every 2-3 days and maintained at 20°C.

Male albino CF-1 mice (18-25g, ~4-5 weeks old; Envigo, Harlan) were used as experimental animals. All animals were allowed free access to food (irradiated Picolab 20 5053, LabDiets) and filtered tap water, except during experimental procedures, and were used only once. *In vivo* antiseizure activity was established through both electrical and chemoconvulsant seizure paradigms. Vehicle-treated mice were also tested to confirm no anticonvulsant activity of the formulation vehicle. All rodent studies were performed at and approved by the University of Washington Institutional Animal Care and Use Committee and conformed to the ARRIVE Guidelines[3].

Compound Library Screening in zebrafish

The Johns Hopkins Clinical Compound Library (JHCCL;[4]) was stored with each compound at a concentration of 5 mM in DMSO in 25 v-bottomed 96-well microtitre plates (Matrix) at –80°C. Assay plates contained compounds diluted to 25 µM in E3 media for drug screening. For the anticonvulsant assay, embryos were raised to 50 hpf and treated with Pronase (Sigma) to remove the chorions. Embryos were aliquoted at three or four embryos per well into Multiscreen mesh-bottomed plates (100 mm; Millipore) and transferred to Multiscreen 96-well culture receiver trays (Millipore) containing the JHCCL compounds at 25 µM in columns 2-11, with control wells containing either 2.5 mM VPA, E3 or DMSO only in columns 1 and 12.

Assay plates were incubated in the dark at 28°C for 90 minutes followed by addition of PTZ to a final concentration of 20 mM to all the compound wells and half of the control wells. Assay plates were incubated for 1 hour at 28°C and the embryos were then transferred to fixative containing 4% PFA and stored at 4°C overnight. Embryos were bleached according to the standard protocol [5] and stored at -20°C in methanol until required for *in situ* hybridization. In order to facilitate screening and eliminate the need to transfer embryos between plates at any stage of the process, samples were maintained in the same 96-well mesh-bottomed Multiscreen plates (Millipore) during drug treatment, *in situ* hybridization and hit detection. Chlorothymol was one of the hits identified in the primary screen and rescreened using the method above. Selected compound stocks were then purchased separately from Sigma and tested in concentration-response assays to further confirm the identity of the selected hit compound.

Induction and analysis of PTZ-induced convulsive behavior in zebrafish and C. elegans.

D. rerio: 3 dpf fish were transferred to 48-well plates for measurement in E3 solution. 20 mM PTZ was added immediately before measurement to the appropriate wells. Fish were pre-incubated in treatment compounds for 90 min before measurements were taken. Fish movements were measured using the Zebrabox/Zebralab (*Zebrabox Viewpoint*) automated locomotion tracking system [6]. Measurements were taken over a 1-hour period with light/dark intervals every 2 min (light driver intensity: 10%). For each embryo group incubated with compounds, the mean distance moved over the total time analysed and at individual time intervals were calculated.

C. elegans were age synchronised using the bleaching technique. Worms were washed off of the plate using 3.5 ml sterile H_2O. 1.5 ml of commercial bleach diluted with 5 M NaOH (2:1) was then added. Following repeated vortexing over a 10-min period, this solution was

centrifuged at 1300 *g* and aspirated to 0.1 ml. Sterile H_2O was used to resuspend the pellet and a second centrifuge/aspiration step performed. Eggs were pipetted onto a freshly seeded NGM plate.

All *C. elegans* PTZ assays were conducted in Dent's solution: 0.81 g NaCl, 0.45 g KCL, 0.20 g $MgCl_2$, 0.12 g Hepes, 1 ml of 1 M $CaCl_2$ and made up to 100 ml with dH_2O (pH to 7.4). BSA was added at 0.06% to prevent worm adherence to the assay plate. Depending on the measurements being conducted, various concentrations of PTZ were dissolved into Dent's solution. For the majority of the experiments conducted, 7 mg/ml of PTZ (50 mM) was used and worms were incubated in this solution for 15 mins before 30 seconds of observation.

Both qualitative and quantitative measurements were performed as previously described[2]. For qualitative data, a yes/no seizing score was determined by if the measured *C. elegans* showed three repetitive convulsions at any time during a 30-second measuring period. Also included in the qualitative data was scoring if worms were paralysed by the treatment (no movement for 30 seconds). A convulsion was defined as an extension and retraction of the head of the worm (an atypical worm behaviour when not presented with PTZ). The number of convulsions was also scored over the 30 seconds to provide a quantitative measure. Worms were excluded from the data if abnormal behaviour was seen such as stiffness or minimal movement determined using touch responsiveness post-recording. This was classified as a potential fatality during the experiment.

Zebrafish qPCR

RNA extraction

Immediately following locomotion assays, 3 dpf fish were isolated for qPCR analysis and snap-frozen on dry ice. All 8 fish were taken per treatment for each repeat creating 3 samples per

group. RNA extraction was conducted using the Trizol/chloroform method. Following RNA extraction samples were measured using nanodrop spectrophotometry for absorbance at 260 nm and 280 nm wavelengths to determine relative RNA concentration.

cDNA synthesis

cDNA was synthesised using *Thermofisher superscript II* reverse transcriptase. 400 ng of RNA sample was combined with 0.5 µl of oligo dT (500 µg/ml), 0.5 µl dNTP (10 mM) which was topped up to 6.5 µl with sterile H_2O. Using heat blocks; samples were heated to 65°C for 5 mins then chilled on ice. Samples were then centrifuged and an additional 2 µl of first strand buffer and 1 µl 0.1 M DTT was added. Following this samples were incubated at 42°C for 50 mins. Reaction was inactivated by heating to 70°C for 15 mins and stored at -70°C.

Quantitative PCR reaction assembly and thermal cycling

cDNA concentration and purity were measured using nanodrop spectrophotometry. 1 µl of 100 ng/µl cDNA template was loaded into each well with 5 µl Sybr green, 0.6 µl FW primer, 0.6 µl RV primer and 2.8 µl H_2O, making a 10 µl total mixture. qPCR mixtures were loaded into *Bio-Rad* 96-well hard shell PCR plates. Thermo-cycling was conducted using the *Bio-Rad* CFX real-time PCR detection system. Temperature was held at 95°C for 3 min, then 40 cycles of the following temperatures; 95°C for 30 seconds, 60°C for 30 seconds and 72°C for 30 seconds. Melting curves were measured through an increase from 65 to 95°C by 0.5°C every 5 seconds.

Gene	Sequence FW	Sequence RV
β-actin 1	CGAGCAGGAGATGGGAACC	CAACGGAAACGCTCATTGC
c-fos	AACTGTCACGGCGATCTCTT	TTGGAGGTCTTTGCTCCAGT

***C. elegans* paralysis assay**

C. elegans were incubated in a 300 µM chlorothymol, 0.1% DMSO and 0.6% BSA solution for 15 min prior to observation for 30 seconds. Worms were scored as having a paralysis phenotype if they exhibited a 10 second period of limited movement. This was defined as an inability of the worm to move either head or tail across the head-tail axis. Worms were given a mechano-sensory stimulus to ascertain survival following observation. For the addition of bicuculline methiodide, it was first dissolved in a stock concentration in 100% DMSO, following which it was diluted to 10 mM for incubation treatment and the assay conducted as above.

***C. elegans* microinjection**

The UBC_f80M224Q and CBGtg9050C11145D fosmid constructs were obtained from *Source-Bioscience,* with CBGtg9050C11145D having been described previously by Gendrel et al[7]. All injections were conducted as follows. Young-adult *C. elegans* were injected with combinations of 15 ng/µl fosmid construct, 5 ng/µl *myo-2*::mCherry and made up to 120 ng/µl with empty vector filler DNA (pBluescript). For each set of injections, three independently derived lines of each transgenic strain were analysed.

Mouse brain slice preparation and electrophysiology.

Mice were euthanised by cervical dislocation in accordance with Schedule 1 of the UK Government Animals (Scientific Procedures) Act 1986. Thalamic slices were prepared from C57/Bl6 mice (P18-24) of either sex according to standard protocols[8]. Briefly, dissected brains were sliced horizontally (320 μm) in an ice-cold sucrose based cutting solution, using a Leica VT1200S vibrating microtome. The slices were incubated at room temperature (20-23 ^{o}C) for a minimum of 1 hour prior to recording in an oxygenated, extracellular solution (ECS) containing (in mM): 126 NaCl, 2.95 KCl, 26 $NaHCO_3$, 1.25 NaH_2PO_4, 2 $CaCl_2$, 10 D-glucose and 2 $MgCl_2$ (pH 7.4; 300-310 mOsm).

Electrophysiological recordings were obtained from ventrobasal (VB) thalamocortical neurons in slices maintained in warmed (35°C) ECS. Cells were visually identified with an Olympus BX51 microscope (Olympus, Southall, UK) equipped with differential interference contrast/infrared optics and a CCD camera. Whole-cell voltage-clamp recordings were obtained using patch pipettes prepared from thick walled borosilicate glass (Garner Glass Company, Claremont, California, USA), and had open tip resistances of 4-5 MΩ when filled with intracellular solution (ICS) that contained (in mM): 135 CsCl, 10 HEPES, 10 EGTA, 2 Mg-ATP, 1 $CaCl_2$, 5 QX-314 (pH 7.3 with CsOH, 300-305 mOsm). Miniature inhibitory post-synaptic currents (mIPSCs) and tonic inhibition were recorded using an Axopatch 200B amplifier (Axon Instruments, Foster City, CA USA) at a holding potential of –60mV in ECS that additionally contained 2 mM kynurenic acid and 0.5 μM tetrodotoxin (TTX) to block ionotropic glutamate receptors and sodium-dependent action potentials, respectively. Series resistance (compensated up to 80%) was monitored intermittently throughout the duration of experiments, with recordings discontinued if values varied by more than 25% or exceeded 20 MΩ. Currents were filtered at 2 kHz, and digitised to PC (10 kHz) using a NI-DAQmx analogue-digital interface for subsequent offline analysis.

Chlorothymol was prepared as a concentrated 10 mg/ml stock solution (1000x) in DMSO and diluted to the final concentration (54 µM) in ECS. The final maximum DMSO concentration (0.1 % v/v) had no effect on mIPSCs, or the tonic current. Chlorothymol was applied via the perfusion system (2-4 ml/min) and allowed to infiltrate the slice for a minimum of 10 min while recordings were acquired. Salts were obtained from VWR and all antagonists from HelloBio (UK).

Whole-cell recordings were analysed offline using the Strathclyde Electrophysiology Software, WinEDR/WinWCP (J. Dempster, University of Strathclyde, UK) software package. Miniature IPSCs were analysed with respect to peak amplitude, 10-90% rise time, charge transfer, and decay time course. The decay time course of mIPSCs was described adequately in all cases by fitting a double exponential function ($y(t) = A_1e^{(-t/\tau 1)} + A_2e^{(-t/\tau 2)}$) using the least squares method, where A is amplitude, t is time and τ is the decay time constant. From the initial bi-exponential fit, a weighted decay time constant (τw) was also calculated according to the equation:

$\tau w = \tau_1 P_1 + \tau_2 P_2$,

where: τ_1 and τ_2 are the decay time constants of the first and second exponential functions and P_1 and P_2 are the proportions of the synaptic current decay described by each component. The magnitude of the tonic current under control conditions and in the presence of chlorothymol was calculated as the difference in the holding current before (in control or in the presence of chlorothymol) and after application of bicuculline methobromide. All results are reported as the mean ± standard error of mean (SEM). Statistical significance of the mean data was assessed in GraphPad Prism v6 with Student's *t* test used to assess paired data.

Human brain slice preparation and electrophysiology.

Human tissue was obtained from neurosurgical patients at Royal Victoria Infirmary, Newcastle, in accordance with ethical approval by the Newcastle and North Tyneside 2 Research Ethics Committee (06/Q1003/51) with clinical governance approval by the Newcastle Upon Tyne NHS Foundation Trust (CM/PB/3707). Human neocortical slices were prepared from fresh brain tissue obtained from the margin of resection from patients that underwent neurosurgery for brain tumours. We included recordings from 6 slices obtained from two patients. The information about the two patients and tissue localization is illustrated below:

Patient number	Gender	Age	Hemisphere	Lobe	Diagnosed Pathology
1	Female	61	Left	Occipital	Glioblastoma
2	Female	53	Right	Frontal	Glioblastoma

After resection, tissue was placed within ~10 s in ice-cold slicing artificial cerebrospinal fluid (aCSF) containing (in mM): sucrose 180, $NaHCO_3$ (25), KCl (2.5), NaH_2PO_3 (1.25), $CaCl_2$ (0.5), $MgSO_4$ (10), glucose (10), ethyl-pyruvate (20), ascorbic acid (1), NAC (2), taurine (1), indomethacin (0.0445), and aminoguanidine (0.148), 290-310 mOsm, continuously saturated with 95% O_2 and 5% CO_2. Samples were transported to the lab in sealed conditions for 10 minutes. Pia and surface blood vessels that would obstruct slicing were removed with forceps and slicing was performed with a vibrating blade microtome (Microm HM 650V) in ice-cold slicing aCSF. 300-μm slices were incubated for ~30 minutes at room

temperature in holding aCSF containing (in mM): NaCl (126), KCl (3), $NaHCO_3$ (24), NaH_2PO_3 (1.25), $CaCl_2$ (2), $MgSO_4$ (2), glucose (10), 290-310 mOsm, saturated with 95% O_2 and 5% CO_2. For recordings, the slices were moved to the electrophysiology rig and perfused with recording aCSF containing (in mM): NaCl (126), KCl (3), $NaHCO_3$ (24), NaH_2PO_3 (1.25), $CaCl_2$ (1.2), $MgSO_4$ (1), glucose (10), 290-310 mOsm, saturated with 95% O_2 and 5% CO_2. Recordings were performed up to 14 hours after tissue resection.

GABAergic inhibitory currents were isolated and recorded by using voltage clamp mode recordings at a holding membrane potential of +10 mV, which is close to the reversal potential for glutamate [9]. All recordings were performed using an Axopatch 200B amplifier and WinWCP Strathclyde Whole Cell Analysis software (University of Strathclyde) and stored for off-line analysis. Patch pipettes of 3–5 MOhM tip resistance were pulled from filamental borosilicate glass capillaries (1.2 mm outer diameter, 0.69 mm inner diameter; Harvard Apparatus), using a horizontal puller (Sutter P-97) and filled with internal solution. The composition of internal solution for voltage clamp recordings was (in mM): K-gluconate (130), NaCl (10), Na_2ATP (3.5), Na_3GTP (1), $MgCl_2$ (2), EGTA (1), QX-314 (0.2) and HEPES (10). Given the low intracellular Cl^- concentration and holding current of +10 mV, inhibitory currents are recorded as positive deflections in the traces (Figure 4B,C). This method allows for recording inhibitory currents experienced by a neuron without altering the activity of the network.

In order to isolate and calculate the individual contribution of phasic and tonic inhibitory currents (I_{Pha} and I_{Ton}) we used a method previously developed and described by Glykys and Mody[10]. In brief, the recordings were split into 30 seconds long epochs, an all-point histogram was constructed for each 30 seconds epoch for the distribution of inhibitory currents, and a Savitzky–Golay filter was used to smooth the histogram and determine its peak. A Gaussian was then fitted to the side of the histogram more negative than the peak, which is

un-affected by the phasic events. All points within the Gaussian fit were classified as part of the tonic current (I_{Ton}), while points more positive than a threshold (set at the peak of the Gaussian + 3 SDs), were classified as part of the phasic current (I_{Pha}). We used 2-minute periods before and after different drug manipulations to test the effects of drugs on I_{Pha} and I_{Ton}. The peak of the Gausian was considered as the mean holding current and I_{Ton} was calculated as the difference in holding current before and after drugs manipulation[10]. I_{Pha} was calculated as the mean of all the recording points after subtraction of the holding current from the recording, which brings the mean of the Gaussian to 0 and thus captures only the contribution of I_{Pha}[10].

We tested the effect of chlorothymol on I_{Pha} and I_{Ton} by sequentially applying chlorothymol (54 µM). We initially applied chlorothymol and recorded the change in holding current and the change in I_{Pha} induced by chlorothymol. We next washed in bicuculine, which blocks both phasic and tonic GABAergic currents. This allowed us to calculate the absolute I_{Ton} as the differences in holding current between baseline conditions and during bicuculline treatment. The effect of chlorothymol on I_{Ton} was calculated as percentage change relative to the absolute I_{Ton}.

Data was analysed using custom made scripts in Matlab R2017B and presented as mean ± standard error of the mean (SEM).

Mouse in-vivo seizure and motor impairment tests.

Mice were prepared for electrically-evoked seizures with application of a drop of anaesthetic/electrolyte solution (0.5% tetracaine hydrochloride in 0.9% saline) to the eyes before placement of bilateral corneal electrodes. A chlorothymol stock (25 mg/ml) was prepared in 40% hydroxypropyl-β-cyclodextrin (HPBCD) solution for IP administration. For MES test, the electrical stimulus was 50 mA, 60 Hz for 0.2 sec delivered using equipment

similar to that described by Woodbury and Davenport[11]. Absence of tonic hindlimb extension was considered protected. Mice were challenged with 6 Hz stimulation used to induce seizures at 32 mA and 44mA for a duration of 3 sec via corneal electrodes. 32 mA and 44 mA are 1.5 times and 2 times the CC97 of male CF-1 mice (current intensity that evokes seizure in 97% of the population tested[12]). Typically, 6 Hz seizures are characterised by an initial momentary stun followed immediately by forelimb clonus, twitching of the vibrissae, and Straub tail. Animals not displaying this behaviour were considered protected. The subcutaneous PTZ chemoconvulsant seizure assay was conducted in mice with a dose of 85 mg/kg dissolved in 0.9% saline, the convulsant dose of 97% of male CF-1 mice[12]. Animals were observed for 30 mins for the presence of a 3-sec clonic episode. Absence of a seizure in the 30-min observation period was scored as protection.

For corneal kindling, mice were kindled to a criterion of 5 consecutive secondarily generalized seizures (Stage 4 or 5, as described by Racine[13]) according to the corneal kindling protocol previously described[14; 15]. Twice daily, 0.5% tetracaine was applied to each eye and the optic nerve was stimulated through bilateral corneal electrodes (3 mA, 60 Hz, 3 seconds). Each stimulation session was separated by at least 4 hours. After receiving twice daily corneal stimulations, CF-1 mice typically reached the first Stage 5 seizure within approximately 8-10 days. Twice daily stimulations continued for each mouse until that animal had achieved the criterion of 5 consecutive Stage 5 seizures, whereby it was considered "fully kindled", which typically occurs after 10-14 days. Fully kindled mice were then stimulated once every other day until all other mice within the group achieved the criterion of 5 consecutive Stage 5 seizures. Testing of chlorothymol commenced at least 5-7 days after the last stimulation necessary to achieve kindling criterion for all mice in the group[14]. Quantitative differentiation studies were performed 1 hour after drug dosing (at the TPE). Mice displaying a seizure score

$\leq$ 5 were considered protected. Each corneal kindled mouse was allowed at least 3-4 days between tests to washout any investigational compound after testing.

The fixed-speed rotarod test was used to establish minimal motor impairment (MMI)[16]. When a mouse is placed on a 1-inch knurled rod that rotates at a speed of 6 r.p.m., the animal can maintain its equilibrium for sustained periods of time. Compounds were considered toxic if treated mice fell off this rotating rod three times during a 1-minute period.

Statistical analysis

Phenotype analysis: Statistical analysis was generally performed using *GraphPad Prism version 6* (Graphpad Software Inc, California USA) using Student's *t* tests or ANOVA with appropriate corrections for multiple testing. For mouse phenotyping; time of peak efficacy (TPE) of chlorothymol was first established in n = 4 mice for all acute seizure tests. All quantitative *in vivo* antiseizure/behavioral impairment studies were then conducted at the TPE of that seizure test. For all acute seizure tests, groups of at least n = 8 mice were tested with various doses of chlorothymol until at least 2 points were established between the limits of 100% protection or MMI and 0% protection or MMI. The dose of drug required to produce the desired endpoint in 50% of animals (ED_{50} or TD_{50}) in each test, the 95% confidence interval and the slope of the regression line were then calculated by Probit analysis[17].

qPCR analysis: The fold change in gene expression for qPCR experiments was calculated using the delta-delta CT quantification method. *c-fos* fold change was normalised against actin

as a housekeeping gene. Larvae from each treatment group were pooled, giving 3 samples of 8 larvae. Data shown are the mean of the 3 samples with SEM.

References

1. Westerfield M. The Zebrafish Book. A Guide for the Laboratory Use of Zebrafish (Danio rerio). University of Oregon Press, Eugene; 2007.
2. Wong SQ, Jones A, Dodd S, Grimes D, Barclay JW, Marson AG, et al. A Caenorhabditis elegans assay of seizure-like activity optimised for identifying antiepileptic drugs and their mechanisms of action. *J Neurosci Methods* 2018;309:132-142.
3. Drummond GB, Paterson DJ, McGrath JC. ARRIVE: new guidelines for reporting animal research. *J Physiol* 2010;588:2517.
4. Chong CR, Chen X, Shi L, Liu JO, Sullivan DJ, Jr. A clinical drug library screen identifies astemizole as an antimalarial agent. *Nat Chem Biol* 2006;2:415-416.
5. Thisse C, Thisse B. High-resolution in situ hybridization to whole-mount zebrafish embryos. *Nat Protoc* 2008;3:59-69.
6. Baxendale S, Holdsworth CJ, Meza Santoscoy PL, Harrison MRM, Fox J, Parkin CA, et al. Identification of compounds with anti-convulsant properties in a zebrafish model of epileptic seizures. *Dis Model Mech* 2012;5:773-784.
7. Gendrel M, Atlas EG, Hobert O. A cellular and regulatory map of the GABAergic nervous system of C. elegans. *Elife* 2016;5.
8. Belelli D, Peden DR, Rosahl TW, Wafford KA, Lambert JJ. Extrasynaptic $GABA_A$ receptors of thalamocortical neurons: a molecular target for hypnotics. *J.Neurosci.* 2005;25:11513-11520.

9. Jirsa VK, Stacey WC, Quilichini PP, Ivanov AI, Bernard C. On the nature of seizure dynamics. *Brain* 2014;137:2210-2230.

10. Glykys J, Mody I. The main source of ambient GABA responsible for tonic inhibition in the mouse hippocampus. *J Physiol* 2007;582:1163-1178.

11. Woodbury LA, Davenport VD. Design and use of a new electroshock seizure apparatus, and analysis of factors altering seizure threshold and pattern. *Arch Int Pharmacodyn Ther* 1952;92:97-107.

12. Barker-Haliski M, Harte-Hargrove LC, Ravizza T, Smolders I, Xiao B, Brandt C, et al. A companion to the preclinical common data elements for pharmacologic studies in animal models of seizures and epilepsy. A Report of the TASK3 Pharmacology Working Group of the ILAE/AES Joint Translational Task Force. *Epilepsia Open* 2018;3:53-68.

13. Racine RJ. Modification of seizure activity by electrical stimulation. II. Motor seizure. *Electroencephalogr Clin Neurophysiol* 1972;32:281-294.

14. Rowley NM, White HS. Comparative anticonvulsant efficacy in the corneal kindled mouse model of partial epilepsy: Correlation with other seizure and epilepsy models. *Epilepsy Res* 2010;92:163-169.

15. Matagne A, Klitgaard H. Validation of corneally kindled mice: a sensitive screening model for partial epilepsy in man. *Epilepsy Res* 1998;31:59-71.

16. Dunham NW, Miya TS. A note on a simple apparatus for detecting neurological deficit in rats and mice. *J Am Pharm Assoc Am Pharm Assoc* 1957;46:208-209.

17. Finney DJ. Probit Analysis. Cambridge University Press: London; 1971.

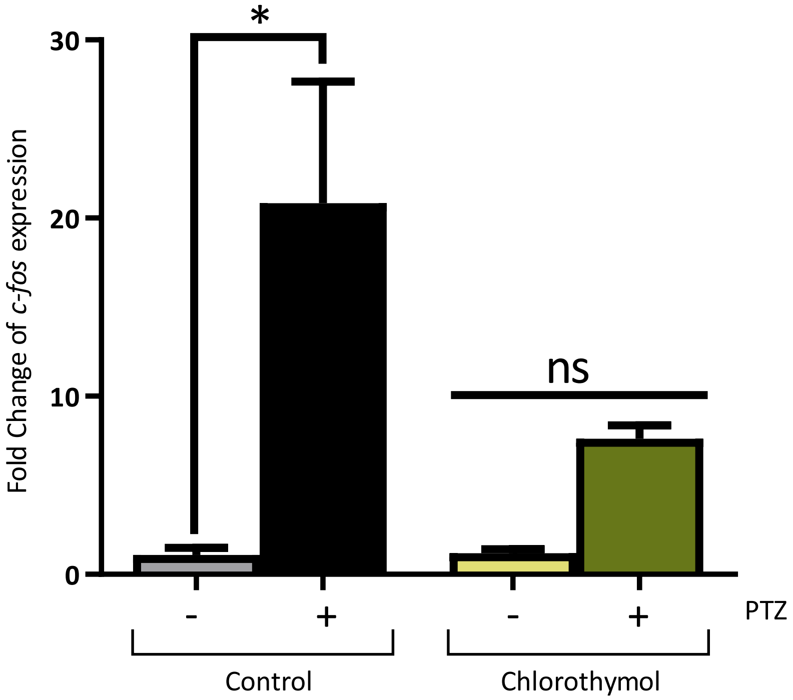

Supplementary Fig S1. Chlorothymol inhibits PTZ-induced c-fos expression in zebrafish.
Larvae at 3 d.p.f. were exposed to either DMSO vehicle only (CTRL) or 25 µM chlorothymol in DMSO before exposure to 20 mM PTZ. Quantitative real-time PCR analysis was then used to determine the relative expression level of c-fos mRNA. Data displayed show mean values ± SEM (n=8 fish per experiment; N=3 independent experiments). Data were analysed using one-way ANOVA with Tukey's multiple comparison test (* P<0.05 compared to PTZ-treated animals).

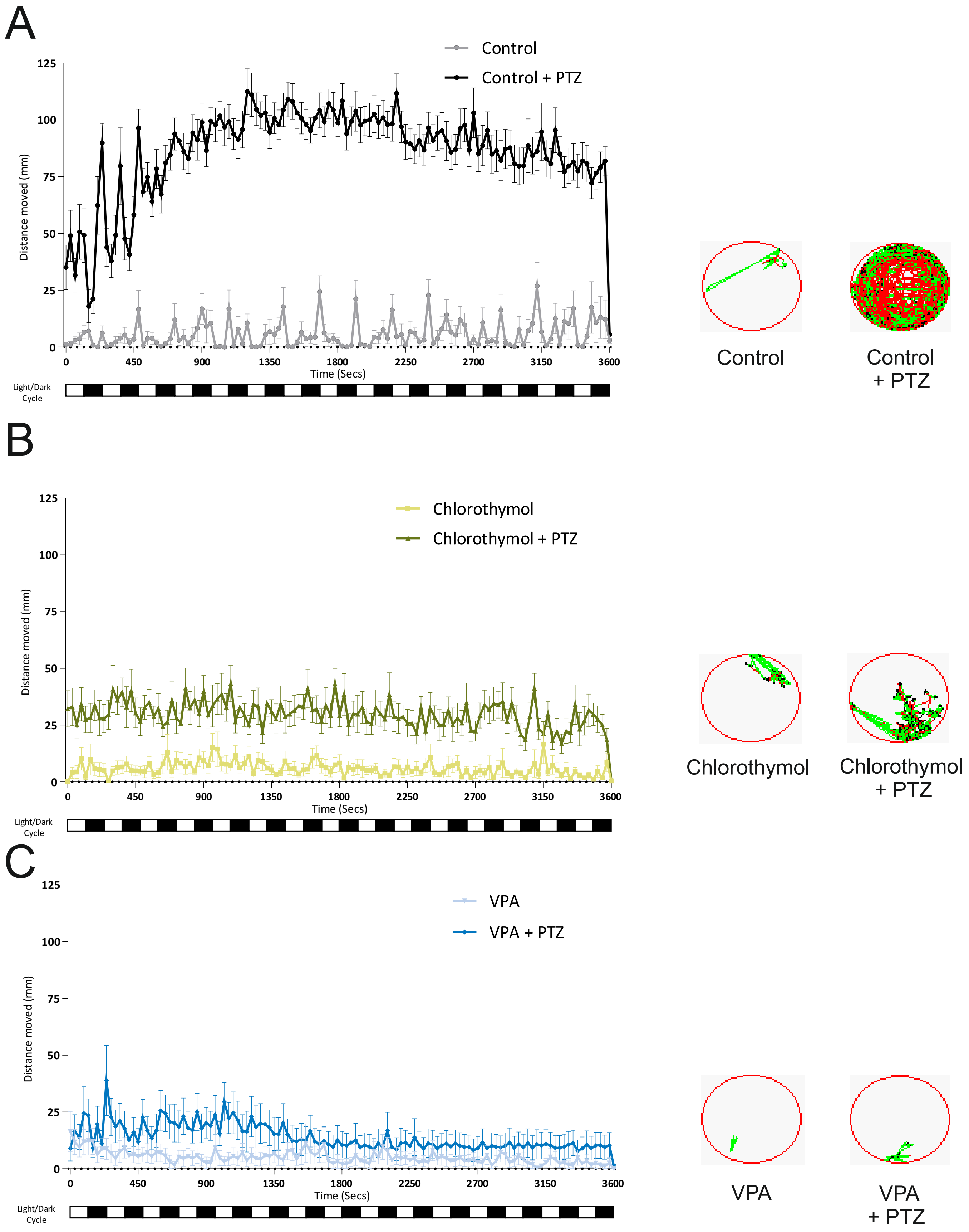

Supplementary Fig S2. Chlorothymol reduces seizure-like behaviour in zebrafish.
Automated video tracking was used to record movement of 3 d.p.f. larvae. Panels on the left show individual time-points following PTZ application (time-point 0). The black and white ruler displays alternating light/dark intervals used. Panels on the right show example well traces of fish movement over the recording period. Black, green and red traces indicate swimming speeds of 0–1.5, 1.5–6 and >6 mm/second, respectively. (A) Data from fish treated with 20 mM PTZ only, (B) fish pre-treated with 25 μM chlorothymol and (C) fish pre-treated with 2.5 mM VPA. Data displayed show mean values ± SEM (n=24 fish per experiment; N=3 independent experiments).

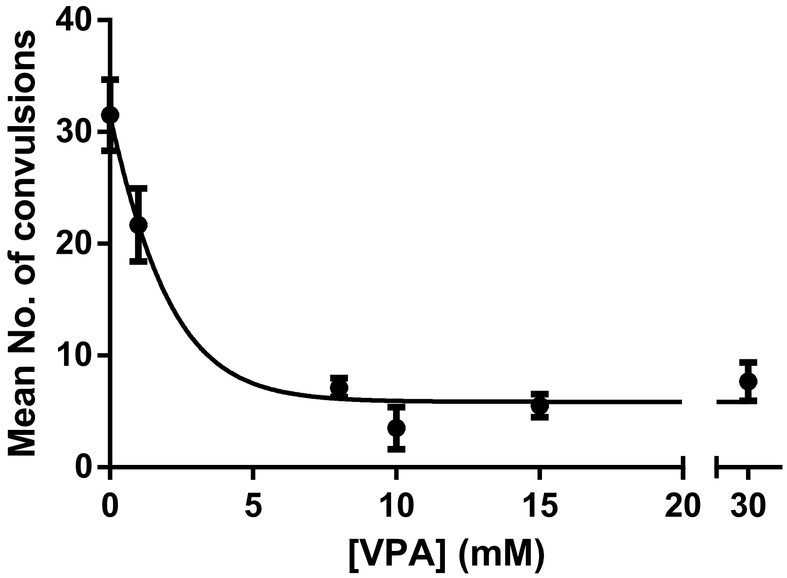

Supplementary Fig S3. Determination of the optimal anticonvulsant VPA concentration in *C. elegans*.

Seizure-prone *unc-49(e407)* mutant worms were pre-incubated for 2 hours in the presence of the indicated concentrations of VPA. They were then transferred to droplets containing 50 mM PTZ and the same concentration of VPA. The total number of head-bobbing convulsions was measured over a 30-second period and displayed as a % of PTZ-treated controls ± SEM (n=10 worms per experiment; N=3 independent experiments).

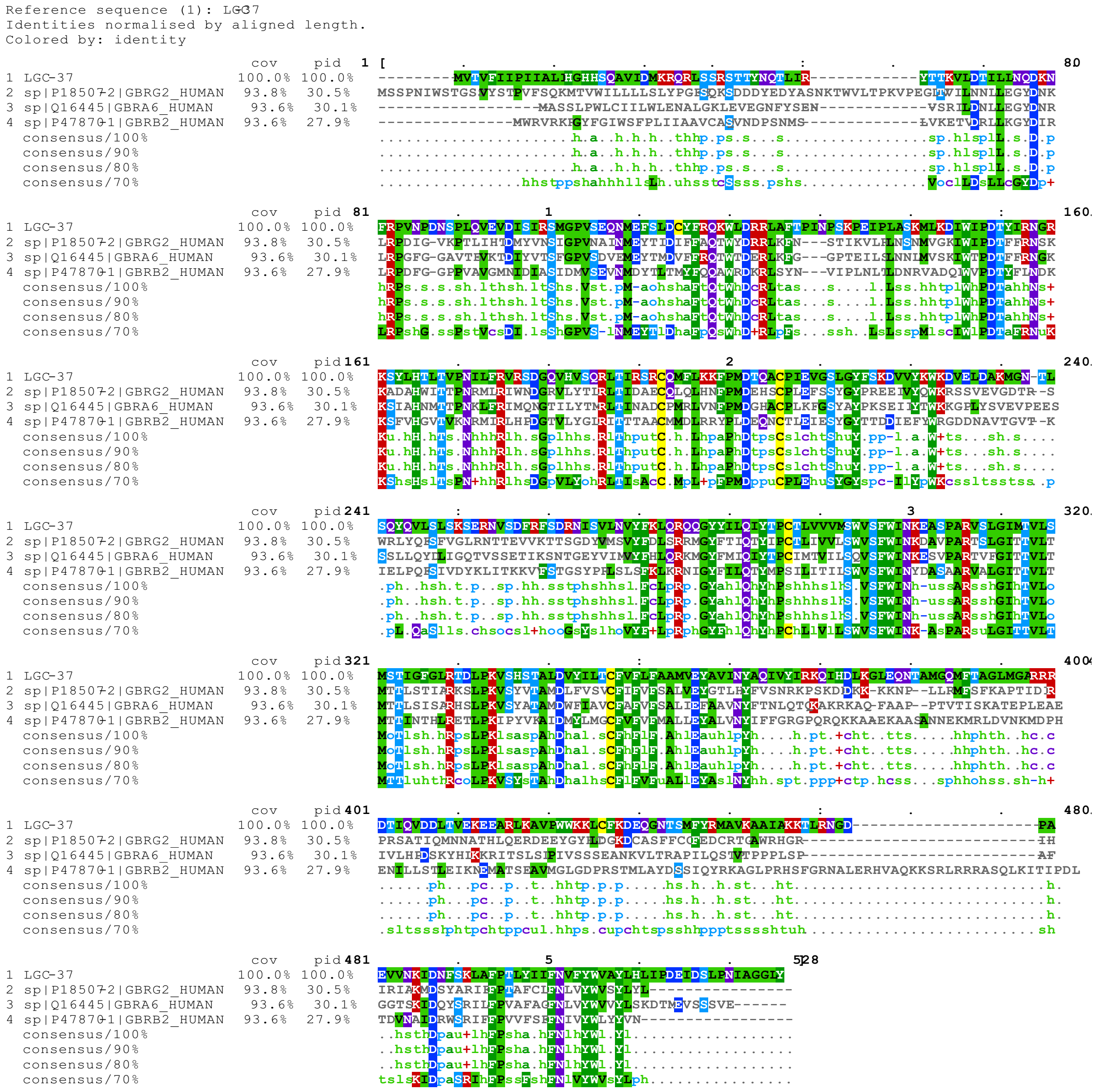

Supplementary Fig S4. LGC-37 is a worm GABA$_A$ receptor subunit.

Clustal Omega alignment of the amino acid sequence of *C. elegans* LGC-37 with its closest human homologues by BLASTP: the GABA$_A$ receptor gamma 2, alpha 6, and beta 2 subunits.

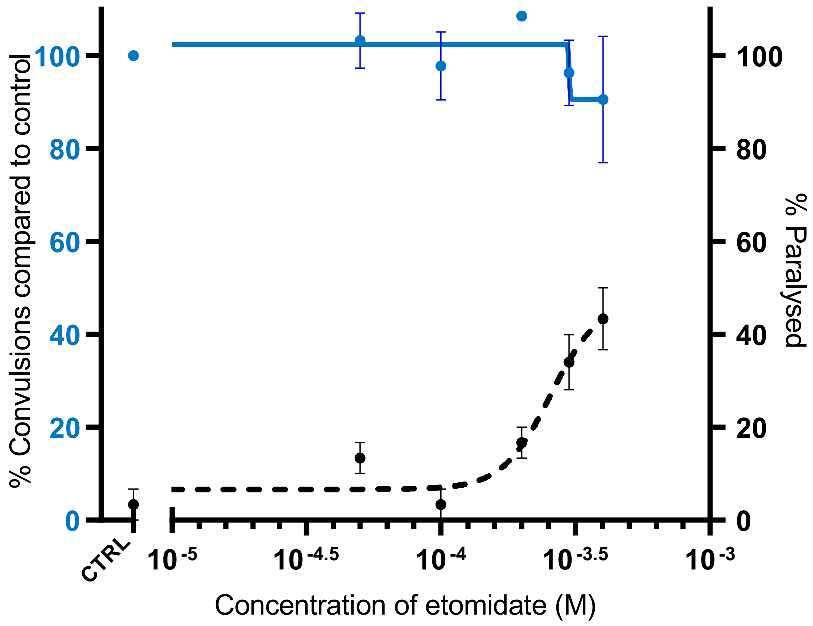

Supplementary Fig S5. Etomidate has no anticonvulsant activity in *C. elegans*.
Seizure-prone *unc-49(e407)* mutant worms were pre-incubated with a range of etomidate concentrations in the presence or absence of 50 mM PTZ. The total number of head-bobbing convulsions in the presence of PTZ was measured over a 30-second period and expressed as a percentage of PTZ-treated controls (blue line). The proportion of paralysed worms in the absence of PTZ was also determined (black dotted line). Data are displayed as mean ± SEM (n=10 worms per experiment; N=3 independent experiments).

	Control	Chlorothymol
**mIPSC peak amplitude (pA)**	-43.3 ± 2.2	-51.1 ± 3.1**
**mIPSC rise time (ms)**	0.51 ± 0.05	0.53 ± 0.03
**mIPSC charge transfer (fC)**	-161.8 ± 13.8	-293.7 ± 38.6**
**mIPSC τw (ms)**	3.6 ± 0.2	5.8 ± 0.4***
**Tonic Current (pA)**	-32.4 ± 11.1	-58.9 ± 15.7*

Supplementary Fig. S6: Chlorothymol increases GABAergic activity in mouse brain slices. The effect of bath application of 50 µM chlorothymol on miniature inhibitory post-synaptic currents (mIPSCs) and tonic inhibition in mouse thalamic VB neurons was recorded. Data shown are mean ± SEM (n=2 brain slices per experiment; N=3 independent experiments) and were analysed by paired t-test (* $P<0.05$, ** $P<0.01$, *** $P<0.001$).

	Control	Chlor othymol
**mIPSC peak ampli tude (pA)**	43.5 ± 10.6	51.2 ± 10.0 *
**mIPSC rise time (ms)**	1.13 ± 0.16	1.13 ± 0.11
**mIPSC charge transfer (pC)**	305.6 ± 70.3	459.1 ± 68.1 *
**mIPSC decay (ms)**	7.18 ± 1.09	7.45 ± 0.91

Supplementary Fig. S7: Chlorothymol increases GABAergic activity in human brain slices. The effect of bath application of 50 µM chlorothymol on miniature inhibitory post-synaptic currents (mIPSCs) in human cortical neurons was recorded. Data shown are mean ± SEM (n=6 brain slices) and were analysed by paired t-test (* P<0.05).

Dose	Result	Individual scores	Average seizure score
30	0/7	5,5,5,5,5,5,5	5 ± 0
50	2/8	5,5,5,2,2,5,5,5	4.25 ± 0.5
75	2/8	1,5,3,2,5,3,5,4	3.5 ± 0.5
150	5/8	0,1,5,5,1,0,5,0	2.13 ± 0.9
200	6/8	0,0,1,5,5,0,1,0	1.5 ± 0.8

Supplementary Fig. S8: Chlorothymol provides dose-dependent anticonvulsant activity in the corneal kindled mouse model of chronic network hyperexcitability. Racine scores for each individual mouse and average scores ± SEM for all mice are shown. Protection result data is shown as n/f, where n is the number of animals protected and f is the total number of mice. A value of 2 or less for an individual mouse is considered protected.
